# Experimental and Theoretical Investigation of Multispecies Oral Biofilm Resistance to Chlorhexidine Treatment

**DOI:** 10.1038/srep27537

**Published:** 2016-06-21

**Authors:** Ya Shen, Jia Zhao, César de la Fuente-Núñez, Zhejun Wang, Robert E. W. Hancock, Clive R. Roberts, Jingzhi Ma, Jun Li, Markus Haapasalo, Qi Wang

**Affiliations:** 1 Division of Endodontics, Department of Oral Biological and Medical Sciences, Faculty of Dentistry, University of British Columbia, Vancouver, British Columbia, V6T 1Z3 Canada; 2 Department of Mathematics, University of South Carolina, Columbia, SC 29208, USA; 3 Centre for Microbial Diseases and Immunity Research, Department of Microbiology and Immunology, University of British Columbia, Vancouver, V6T 1Z3 Canada; 4 Synthetic Biology Group, MIT Synthetic Biology Center, Research Laboratory of Electronics, Department of Biological Engineering, Department of Electrical Engineering and Computer Science, Massachusetts Institute of Technology, Cambridge, MA 02139, USA; 5 Department of Oral Biological and Medical Sciences, Faculty of Dentistry, University of British Columbia, Vancouver, British Columbia, V6T 1Z3 Canada; 6 Department of Stomatology, Tongji Hospital, Tongji Medical College, Huazhong University of Science and Technology, Wuhan, 430030 China; 7 School of Mathematical Sciences, Nankai University, Tianjin 300071, China; 8 Beijing Computational Science Research Center, Beijing 100193, China and School of Materials Science and Engineering, Nankai University, Tianjin 300071, China

## Abstract

We investigate recovery of multispecies oral biofilms following chlorhexidine gluconate (CHX) and CHX with surface modifiers (CHX-Plus) treatment. Specifically, we examine the percentage of viable bacteria in the biofilms following their exposure to CHX and CHX-Plus for 1, 3, and 10 minutes, respectively. Before antimicrobial treatment, the biofilms are allowed to grow for three weeks. We find that (a). CHX-Plus kills bacteria in biofilms more effectively than the regular 2% CHX does, (b). cell continues to be killed for up to one week after exposure to the CHX solutions, (c). the biofilms start to recover after two weeks, the percentage of the viable bacteria recovers in the 1 and 3 minutes treatment groups but not in the 10 minutes treatment group after five weeks, and the biofilms fully return to the pretreatment levels after eight weeks. To understand the mechanism, a mathematical model for multiple bacterial phenotypes is developed, adopting the notion that bacterial persisters exist in the biofilms together with regulatory quorum sensing molecules and growth factor proteins. The model reveals the crucial role played by the persisters, quorum sensing molecules, and growth factors in biofilm recovery, accurately predicting the viable bacterial population after CHX treatment.

Apical periodontitis is an inflammatory reaction of periradicular tissues caused by a microbial infection in the root canal^1^. Because bacteria in the necrotic root canal system grow mostly in sessile biofilms, the success of endodontic treatment depends to a great extent on the effective eradication of such biofilms^2^. Chemo-mechanical instrumentation has been regarded as the key element of endodontic treatment. Mechanical canal preparation supports disinfection by disturbing or detaching biofilms that adhere to canal surfaces and by removing a layer of infected dentin to create some space for disinfecting solutions. Anatomic complexities represent physical constraints that pose a challenge to adequate root canal disinfection. Several studies using advanced techniques such as microcomputed tomography scanning have demonstrated that proportionally large areas of the main root-canal wall remain untouched by the instrument^3^, emphasizing the need for chemical means of cleaning and disinfecting all areas of the root canal. However, the available irrigants that exhibit direct antibacterial activity also face great challenges in eradicating root canal biofilms. The protective mechanisms underlying biofilm antimicrobial resistance are not yet fully understood, although several mechanisms have been proposed. These mechanisms include physical or chemical diffusion barriers to antimicrobial penetration into the biofilm^4^, slow growth of the biofilm due to nutrient limitation, altered gene expression of resistance genes due to activation of general stress responses and/or adaptation to growth on surfaces, and the emergence of a biofilm-specific phenotype^5^.

Endodontic treatment does not always fully eradicate bacteria during infection, leading to interactions between the bacteria and the surrounding host tissues, thus compromising clearance of the infection. Persistent and recurrent apical periodontitis have been the focus of interest in endodontic research for a long time^6^,^7^,^8^,^9^. The primary cause of post-treatment apical periodontitis is acknowledged to be the continuing presence of bacteria within the root canal system^9^,^10^,^11^,^12^. Histopathological investigations found biofilm structures in the great majority (74%) of cases of post-treatment apical periodontitis^13^. Thus biofilms are strongly associated with persistent infection in the root canal.

Irrigation has a central role in endodontic treatment^14^. Several irrigating solutions have antimicrobial activity and can actively kill bacteria and yeasts when in direct contact with the microorganisms^15^,^16^,^17^. The cationic bisbiguanide N,N’1,6-hexanediyl-bis [N’-(4-chlorophenyl) imidodicarbonimidic-diamide] (chlorhexidine digluconate; CHX) is one of the most commonly used irrigant solutions in the clinic due to its antimicrobial properties. CHX is also the active ingredient in many commercially-available disinfectants and antiseptics. As CHX is cationic it interacts with the negatively charged bacterial cell surface and translocates to the cytoplasmic membrane where it damages the membrane barrier leading to cell death^14^,^18^. A 0.2% CHX solution is widely used as an antimicrobial agent to prevent biofilm growth on tooth surfaces. For the current study, we used much higher concentrations to evaluate the susceptibility of dental biofilms to this agent.

Mathematical modeling has emerged as a powerful tool for studying biofilm dynamics; it utilizes a set of experimentally identified or implicated mechanisms and sheds light on how these basic mechanisms can regulate and influence the formation and evolutionary dynamics of biofilms^19^. Mathematical models come in many forms ranging from simple empirical correlations to sophisticated mechanistic, physics-based and computationally intensive ones that can describe three-dimensional biofilm morphology and interaction with the environment^20^,^21^,^22^. Most biofilm models available today, however, capture only a small fraction of the complexities of the biofilm system since each is developed based on a set of idealistic mechanisms, which perhaps apply only to specific biofilm systems. Furthermore, none is able to explain well the dynamic process of multispecies oral biofilm during recovery after treatment with CHX. Therefore, there is an urgent need to develop a new mathematical model to interpret our experimental findings for multispecies oral biofilms.

In this paper we integrate mathematical modeling with an experimental approach to explore the mechanism and the parameters influencing viability of bacteria in biofilms over time after being treated with CHX. Our mathematical model is based on a set of pertinent assumptions regarding biofilm tolerance mechanisms, including the existence of persister cells (which represent dormant or slow growing bacteria in a population that resist the action of antimicrobial agents)^23^ a portion of bacteria that are susceptible to the antimicrobial agent, growth factor molecules and the quorum sensing effect^24^,^25^. It is worth noting that biofilm bacteria become adaptively resistant to antibiotics (resistance determined by the switch to the biofilm life style), but these adaptively resistant organisms that might impact on susceptibility to the given concentrations of antimicrobial agents would be accounted for within the persister population. The model is then calibrated using the well-controlled experiments, making it possible to model the transition between the susceptible and persister cells. In these experiments, we described the effects exerted over time on a multispecies oral biofilm of two preparations containing high levels of CHX. Confocal laser scanning microscopy (CLSM) using fluorescent indicators of membrane integrity (*Bac*Light LIVE/DEAD viability stain) was used to determine biofilm architecture and the proportion of viable bacterial cells within the biofilm. Subsequently, scanning electron microscopy (SEM) was used to visualize compromised bacterial cells resulting from the CHX treatment. The experimental data were then used to calibrate the mathematical model. With the model, we examined the survival of bacteria in biofilms over time after exposure to the CHX protocol, and predicted the populations of bacterial persisters, extracellular polymeric substances (EPS), and quorum sensing (QS) molecules beyond the experimental time scale.

## Results

### Experimental Results

**Figure 1:**
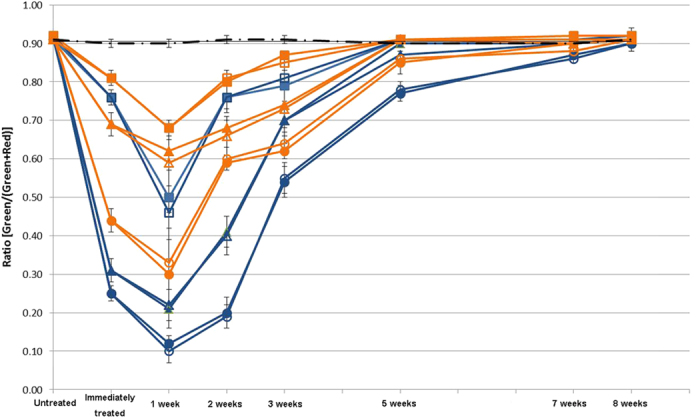
Percentage of live cells in a multispecies biofilm over time. A 3-week-old biofilm was treated for 1, 3 and 10 minutes with the compounds indicated in the graph, respectively. The y-axis ratio corresponds to [live bacteria/(total bacteria)]. Two microliters of inactivator were added for 60 seconds immediately after each 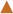 treatment. (**—** • — No Treatment, 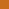 CHX 1 minute with inactivator, 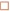 CHX 1 minute without inactivator, CHX 3 min with inactivator, 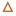 CHX 3 minutes without inactivator, 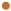 CHX 10 min with inactivator, 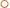 CHX 10 min without inactivator; 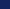 CHX-Plus 1 minute with inactivator, 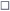 CHX-Plus 1 minute without inactivator, 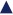 CHX-Plus 3 minutes with inactivator, 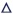 CHX-Plus 3 minutes without inactivator, 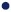 CHX-Plus 10 minutes with inactivator, ○ CHX-Plus 10 minutes without inactivator).

Staining of cells using the LIVE/DEAD stain enabled us to determine the proportion of live-to-dead cells in the biofilm over time. First, biofilms were grown for a period of three weeks and the ratio of live to dead cells was calculated. The mature biofilms were then treated for 1, 3 or 10 minutes with either 2% CHX or CHX-Plus (Fig. 1). Immediately after treatment, the viability profile of the biofilm population changed, demonstrating an increased number of dead cells (Fig. 1). This occurred in all groups, but was more pronounced in the biofilms treated with 2% CHX for 10 minutes and CHX-Plus for 3 and 10 minutes. CHX-Plus showed higher levels of bactericidal activity at all exposure times compared to 2% CHX (*P* < 0.001; Fig. 1) and indeed treatment with CHX-Plus for 3 minutes resulted in greater bacterial cell killing than the treatment for 10 minutes using 2% CHX (Fig. 1). As expected, increasing the treatment time with CHX-plus from 3 to 10 minutes led to increased cell death. The use of a CHX inactivator, which contains L-α-Phosphatidylcholine (α-Lecithin) (Sigma, St. Louis, MO, USA), Tween 80 (Sigma, St. Louis, MO, USA) and distilled water, subsequent to CHX treatment had no significant effect on the viability of the bacterial population, indicating that viability changes were likely due to the exposure to and interaction with CHX during the short application period (Fig. 1). In addition, there were significant differences (*P* < 0.001) with regards to bacterial viability during the recovery phases between the CHX and CHX-Plus irrigants (*P* < 0.001) and after different treatment times (1, 3 and 10 minutes). Cell death in the biofilms continued to increase for up to one week after exposure to the two CHX solutions (*P* < 0.001). Two weeks after the CHX treatment the biofilms had started to recover, as shown by the increase in the ratio of live to total (live + dead) cells (Fig. 1). After five weeks of recovery, the proportion of viable bacteria almost reached the pre-treatment level in all groups (87–91%), but was somewhat less in the 10-minute treatment groups (CHX-Plus: 77%; 2% CHX: 85%) (Fig. 1). Eight weeks after the treatment, bacterial viability in all groups had returned to the pre-treatment level (expressed as percentages).

**Figure 2:**
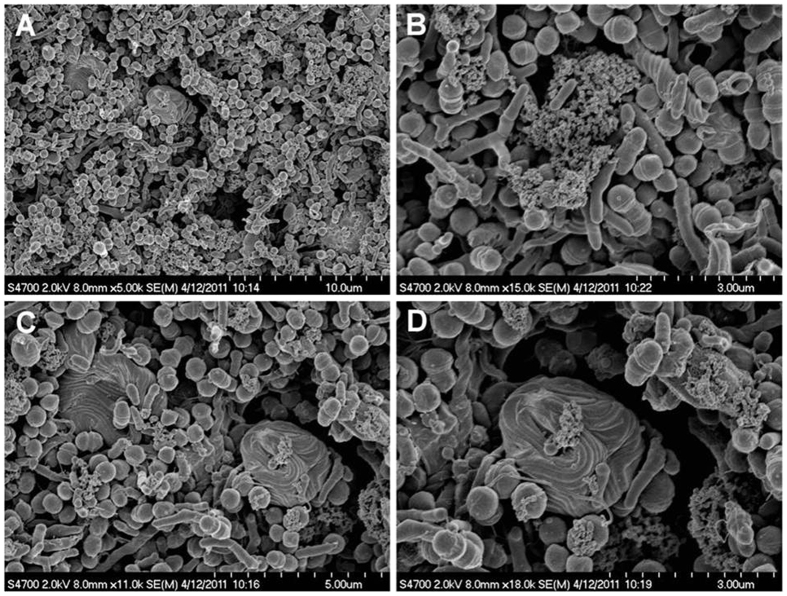
Scanning electron micrograph of a multispecies oral biofilm with mixed bacterial flora including numerous spirochetes. (**A**) three-week old biofilm after being treated with 2% CHX for 3 minutes is shown in (**A**); (**B–D**) higher magnification of (**A**) demonstrating tightly coiled spirochetes and a few damaged bacterial cells.

**Figure 3:**
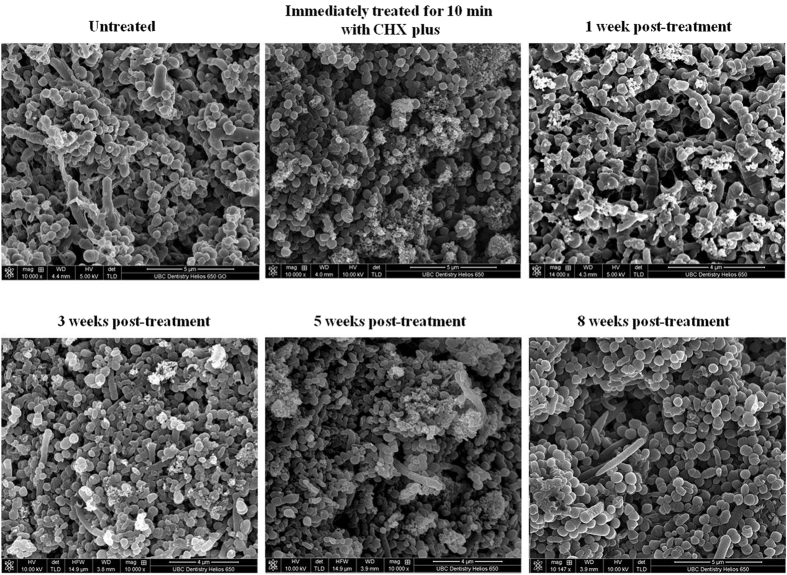
Scanning electron micrographs of a multispecies oral biofilm untreated and treated with CHX-Plus over time.

Scanning electron microscopy (SEM) imaging was also used to visualize the biofilms and to confirm these results. Overall, multispecies in the biofilms were validated by this procedure since cocci, rods and filaments, as well as spirochetes, were present within the biofilms, forming mixed communities (Fig. 2). The untreated samples revealed biofilms that had well organized network structures with virtually no dead or compromised bacterial cells (Fig. 3). However, cell lysis was evident after treatment for 10 minutes with CHX and CHX-Plus, and cell killing was even more pronounced at one week post-treatment (Fig. 3). The number of lysed or damaged cells substantially decreased five weeks after treatment and was practically nonexistent eight weeks after exposure to CHX-Plus (Fig. 3). The thickness of the biofilm decreased from 110 ± 15 μm before treatment to between 53.8 ± 1.8 and 66.0 ± 1.1 μm immediately after the treatments. Contraction in the biofilms continued for one week after the treatments (to between 32.8 ± 3.1 μm and 52.4 ± 6.5 μm) and stopped soon after. The thickness of the biofilm eight weeks after the treatments did not return to its original size and was between 31.8 ± 2.9 μm and 42.2 ± 4.0 μm.

### Numerical Results

#### Mathematical model

**Figure 4:**
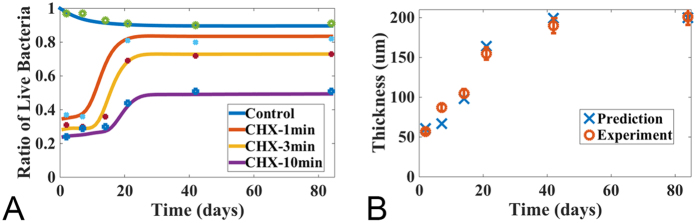
Model prediction: the percentage of the live cell volume of a multispecies oral biofilm treated by CHX and the thickness of a naturally growing biofilm. The initial profile for each components are (0.056, 0.024, 0, 0, 1.0, 0, 0) for the volume fractions of susceptible, persister, live bacteria and EPS, as well as the concentrations for nutrient, antimicrobial agents and QS molecules, respectively. (**A**) Percentage of live bacterial cells in the biofilm at different time after being treated with CHX for 1, 3 and 10 minutes, respectively. The bottom curve corresponds to a controlled experiment where the biofilm is not treated with CHX (experimental data are obtained from^24^); (**B**) Biofilm thickness of the controlled group. The experimental data are plotted with error bars.

**Figure 5:**
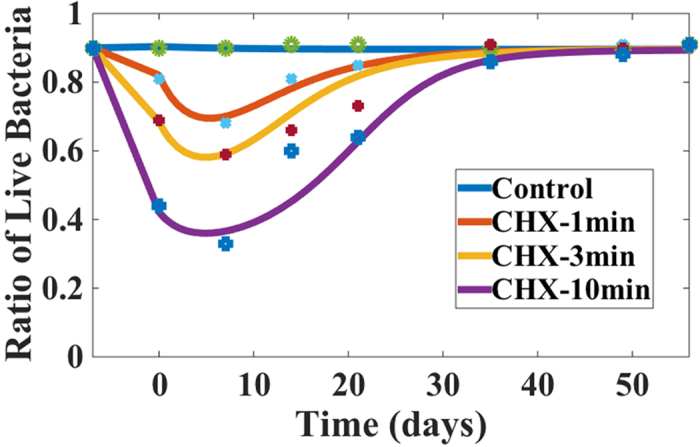
Bacteria in the multispecies oral biofilm after being treated with CHX up to 8 weeks. The initial profile for bacteria components: EPS and QS molecules are from the simulation in Fig. 1 at week 3. For the leftover concentrations of antimicrobial agents, they are fitted using the model as 1.67 × 10^−4^, 2.1 × 10^−4^ and 3.75 × 10^−4^ for CHX 1, 3, 10 minutes treatment correspondingly. The concentration of nutrient is given initially as 1. The percentage of live cell volume in the biofilm during recovery after treatment with CHX for 1, 3, 10 minutes and the control set (without treatment) are shown, respectively. The longer the biofilm is treated with CHX, the longer it takes for the bacterial cells to regain their populations (experimental data are obtained from^26^ and Fig. 1).

We developed a mathematical model to predict the variability of bacteria after antimicrobial treatment. In this model, a conversion mechanism between the susceptible and persister cells, in the absence of selective (antimicrobial) pressure, was assumed in addition to a regulatory role of quorum-sensing molecules and growth factor molecules in bacterial biofilm growth and EPS production. The model was calibrated using the data obtained for the multispecies oral biofilm treated with CHX for 1, 3 and 10 minutes, respectively, at various stages of its natural growth in our previous^26^ and current experiments. Specifically, we calibrated the model parameters to match the dead bacterial population with respect to the three treatments at several selected days when the experimental data were available. One set of the experimental data was plotted in Fig. 1; additional experimental data and the model prediction were plotted in Figs 4 and 5, respectively. The specific values of the model parameters during this calibration are summarized in Table 1^23^,^27^. The model was calibrated to optimize the prediction for the dead bacteria data as well as the live bacteria data from the samples cultured for three weeks before the CHX treatment until eight weeks after the treatment, as well as the dead bacterial population at a set of selected treatment days shown in Fig. 4(A). The coupled ordinary differential equations (ODEs) in the model were solved using a Matlab built-in function, ode45.

#### Antimicrobial efficacy of chlorhexidine against bacteria in biofilms at different stages of development

Using the model, we investigated the persistence of biofilms of different ages in response to antimicrobial agents. In the experiments, biofilms were grown in the lab for up to a maximum of 12 weeks. We calculated the survival proportion, which was defined as the live bacterial volume fraction divided by the total bacterial volume fraction of the bacterial cells, after 1, 3 and 10 minute CHX treatments at various times, as well as for the control set at various ages of grown biofilms. In the same manner, the killing proportion was defined as the dead bactetria volume fraction over the total bacteria volume fraction. The results were shown in Fig. 4(A). These numerical simulations agreed well with the experimental results obtained in our previous study^26^, which demonstrated the applicability and accuracy of the mathematical model.

These analyses revealed that the killing proportion was, in general, a decay function of the biofilm age, i.e., the older the biofilms were, the lower the killing proportion would be. However, three distinct and well-separated regimes for the killing proportion behavior existed with respect to age distribution. High killing proportions were observed for young biofilms, where the killing proportions reached as high as 78% in biofilms treated by CHX for 10 minutes. Even for the biofilm treated by CHX for 1 minute, the killing proportion was as high as 63%. The killing proportion decayed slowly for the biofilms that were less than 10 days old. For biofilms between the age of 10 and 20 days, the killing proportion decayed much more drastically with age. The proportion of killed bacteria was much lower in biofilms 20 days or older than in young biofilms of 2–10 days. After 20 days, the proportion of killed bacteria remained low and relatively unchanged.

The simulation results shown in Fig. 4B indicated that the thickness of the biofilm was proportional to the bacterial volume fraction, consistent with the experimental findings in our previous study^26^. In addition to the results shown, the model is capable of providing more details about the composition of the biofilm. Both the biofilm thickness and the EPS volume fraction reached a plateau after about 24 days when the growth of the bacteria started slowing down; whereas the QS molecules kept increasing until their concentration eventually reached a plateau after about 50 days. Apparently, the increase in the concentration of QS molecules had little impact on the growth of the bacterial population as this growth slowed down between the 25^th^ and the 50^th^ day.

#### Oral multispecies biofilm recovery after CHX treatment

Using the model, we further investigated biofilm recovery after CHX treatment. In particular, we chose the three-week-old biofilm after treatment by CHX, without inactivator, for 1, 3 and 10 minutes, as an example. In the model, we assumed that the post-antibiotic effect was due to residual antimicrobial agents in the biofilm. The numerical results, together with experimental data were depicted in Fig. 5. Quantitatively, the mathematical model agreed well with the experimental data.

The longer the CHX treatment, the longer it took for the biofilm to regain its initial viability levels. The regrowth of the bacterial population correlated with the decrease and depletion of the antimicrobial agents in the biofilm (Fig. 5). Both the susceptible and persistent cell population recovered at specific stages in this study, wherein the population of persistent cells changed because of the on-going conversion between the susceptible and persister cells (Fig. 5).

## Discussion

Endodontic infections can be defined as infections of the pulp and periapical tissues. There are several possible ways by which bacteria may enter the pulp, through caries lesions, enamel and dentin cracks, fractures, open dentin tubules, lateral canals, leaking fillings and rarely also via anachoresis. The multispecies biofilm model developed in the present study does not completely replicate root canal biofilm. However, this model does capture some key characteristics of *in vivo* endodontic biofilms, including the thickness of the biofilm, multispecies nature, and the attachment of bacterial cells to each other. In particular, the collagen-coated hydroxyapatite provides chemical similarity with the teeth/dentin and serves as an excellent substrate for growth of the multispecies biofilm^24^,^25^,^26^,^33^. In the study reported here, this model was used to evaluate the effects of high levels of CHX on biofilms formed by bacterial samples isolated from the subgingival plaque of individual human subjects. We expected to observe extensive cell damage and consequent eradication of the biofilm as a result of the different treatments. Since the primary effect of CHX on bacteria is damage to the cell membrane^34^,^35^, the effect of CHX can be readily visualized by vital staining^24^,^26^,^33^,^36^,^37^. Here we chose to use differential LIVE/DEAD staining rather than CFU counts to assess the ratio of live-to-dead cells over time, since this method has been shown to assess the true viability of cells within biofilms under adverse conditions (such as the presence of CHX) more accurately^25^. The results obtained during the first week post-treatment revealed the expected increase in dead bacterial cells (Fig. 1). However, after the first week of population decline, viable cells increased within the biofilm population and were eventually restored to a mature biofilm level after eight weeks, independent of the treatment (Fig. 1). Confocal laser scanning microscopy imaging confirmed the overall trend observed in viability experiments. These data highlighted the inability of high concentrations of CHX to completely eradicate all bacterial cells in the biofilm, and suggested the presence of a subpopulation of biofilm cells (persisters) capable of tolerating such treatments, that eventually led to a complete recovery of the biofilm.

To better understand the dynamics of the antimicrobial tolerance mechanisms, the mathematical modelling was used to interpret the information gained from *in vitro* experiment in this study. There are several tolerance mechanisms coupled leading to the failure of disinfection of biofilms. The persister population has been estimated to constitute perhaps 0.1–1% of all bacterial cells in a biofilm^38^,^39^. These cells, as hypothesized, can survive a catastrophic antimicrobial challenge and reseed the biofilm; adaptively resistant organisms, while not per se persisters, might further increase the population of tolerant bacterial cells. Biofilms are microbial communities encased by EPS, and engaged in a distinct lifestyle accompanied by a distinct gene expression profile. The EPS matrix provides several functional purposes for the biofilm, such as protecting bacteria from environmental stresses, and providing mechanical stability^40^,^41^. Quorum sensing is a density-dependent cell-cell communication mechanism used by several bacterial taxa to coordinate gene expression and behaviour in groups, based on cell population densities^42^,^43^,^44^. The advantage of mathematical models is that it provides insights into the effect of various factors on the hypothetical mechanisms of antimicrobial biofilms, which thereby minimize the number of experiments needed to investigate complex biological processes. Most previous models have simplified the analysis by focusing on a specific aspect (e.g. the bacterial population, formation of persisters, EPS production or, growth rates and/or mechanisms including QS regulation) in suspended bacteria cultures or during the initial stages of biofilm growth^45^,^46^,^47^,^48^,^49^,^50^,^51^. In the current study, our modeling incorporated each of those factors pertaining to the antimicrobial tolerance mechanisms including the changing bacterial volume fractions of susceptible, persister and dead bacterial cells, the EPS volume fraction, the concentration of the QS molecules, a growth factor molecule, the nutrient concentration and the concentration of antimicrobial agents in oral biofilms at different physiological phases. Biofilms treated by both 2% CHX and CHX-Plus showed very similar susceptibility. Therefore, as an example to explore the dynamics of the tolerance mechanisms, our mathematical model captured the dynamics of biofilms treated by 2% CHX and the subsequent recovery process fairly well.

Drug diffusion, especially of cationic antibiotics, is limited within biofilms due to the presence of an extensive negatively-charged EPS matrix, which hinders the diffusion of CHX into the deepest layers of biofilms. A previous study^26^ showed that the proportion of killed bacteria in mature biofilms was much lower than in young biofilms. This was consistent with our mathematical model, which simulated the dynamic process of oral multispecies biofilm at different ages after treatment with CHX (Fig. 4A). This model was consistent with the conclusion that EPS prevents CHX from penetrating into the biofilm, thus making mature biofilms more difficult to treat compared with young biofilms. In our model, we dealt with this effect as a kinetic issue rather than a spatial diffusive effect of antimicrobial agents. The mathematical model proved to be an effective tool for analyzing the biofilm treatment with CHX. Recently, application of a different mathematical model correctly predicted the ability of QS enabled bacteria to turn on and off the secretion of EPS, thus altering their ability to compete with other species and strains within mixed biofilm^44^. The effects of QS are highly variable and depend both upon the species under observation and the experimental conditions^50^. In the current study, when the thickness of mature biofilms (after 3-weeks of age) reached a “steady state”, EPS production also reached a plateau of accumulation. Conversely, QS molecule concentrations increased for up to 50 days before it reached a steady state. After three-weeks, therefore, QS molecules showed little influence on EPS production. The findings of the current study might help guide researchers to understand the interconnections between biofilm growth, QS and EPS production and how these impact on biofilm development and susceptibility to antimicrobial agents.

Previous studies have shown that treatment with 0.2% CHX significantly inhibits multispecies oral biofilms^36^,^52^. More specifically, exposure to 0.2% CHX induced bacterial cell killing and caused multispecies biofilms to contract at a rate of 1.176 μm.min^−1^ over a period of 15 minutes. It was hypothesized that the mechanism underlying the immediate contraction of oral biofilms upon exposure to CHX was related to ionic interactions between the negatively charged EPS matrix, which comprises the bulk volume of the biofilm, and the positively charged CHX molecules. These interactions would change the hydration, solubility, hydrophobicity, and localized charge along the polymer chains of the EPS. Changes in charge and hydration would in turn affect the tertiary structure of the EPS chains and the degree of bonding with adjacent strands. As the positive CHX interacts with the negative EPS, the net charge of the matrix shifts toward neutral, reducing the repulsive forces between charged moieties, causing glycan chains of the matrix polysaccharides to collapse due to dehydration. These concepts are consistent with the results obtained in the present study, which indicated that the thickness of biofilms decreased by around 50% after treatment with 2% CHX (from 110 μm before treatment to 53–66 μm). Biofilm contraction continued for one week after the treatments, correlating with an increase in dead bacterial cells (Fig. 1).

It was previously shown that as little as 0.02% CHX kills bacterial cells^36^,^52^. In this study, we tested the susceptibility of multispecies oral biofilms to 10-times the concentration of CHX (2% CHX) used previously by Hope and Wilson^53^, in an effort to kill all of the cells present in our multispecies biofilms. Surprisingly, this concentration of CHX was insufficient to fully eradicate oral multispecies biofilms, thus indicating that additional antimicrobial compounds that synergize with CHX might be required to prevent biofilm re-growth in the root canal and in other contexts.

The ability of an antimicrobial to preserve its activity over time is an attractive property, since the concentration of antimicrobial agents tends to drop below the minimal inhibitory concentration over time, thus no longer exerting the desired activity. Importantly, CHX has been shown to exhibit extended residual activity over time, a phenomenon called “substantivity”^54^,^55^ or the “post-antibiotic effect”^56^. The mathematical model predicted that the post-antibiotic effect in the current study might be due to residual antimicrobial agents in the biofilm. Indeed, an *in vivo* study on the mechanism of action of CHX^57^ indicated that CHX inhibits plaque formation by an immediate bactericidal action followed by prolonged bacteriostatic activity of the CHX adsorbed onto the pellicle-coated enamel. Another report evaluating the substantively of 2% CHX within the bovine root canal system, after 10 minutes of application, reported that CHX retained antimicrobial activity even 12 weeks after the initial treatment^58^. Therefore, these previously published results imply that the oral multispecies biofilms presented in this report are quite resistant to high levels of CHX, since the activity of CHX is known to be preserved over a long period of time.

Accurate determination of antimicrobial effectiveness is another important property of an antimicrobial agent. This is measured by the ability of a particular compound to kill microorganisms in a specific amount of time. Hence, appropriate determination of antimicrobial activity requires complete and immediate inactivation (neutralization) of the antimicrobial agent. Ideally, all microorganisms used in evaluating antimicrobial effectiveness should be tested using such an inactivation assay. The effectiveness of inactivation is strongly influenced by the concentration of the active agents, the amount of neutralizing agents, the storage time of the inactivated antiseptic before plating, and by the chosen microorganism. The combination of 3% Tween 80 and 0.3% L-α-lecithin has been found to be the most effective inactivating agent for CHX added to planktonic *E faecalis* and mixed plaque bacteria, allowing full recovery of the test organisms^59^,^60^. In the present study, the use of this inactivating agent had no effect on the activity of the different CHX formulations (Fig. 1), possibly due to its inability to penetrate into the biofilm over the time periods tested.

Our model indicated that the existence of persister cells, which are found in a small portion of the dominant bacteria surviving after CHX antimicrobial treatment, was the main reason for relapse. Persisters would remain in a dormant state promoting tolerance to high concentrations of CHX. However, when the concentration of CHX drops below a threshold at which it was no longer fatal to bacteria, the persisters would become metabolically active, converting back into susceptible cells and beginning to proliferate. Thus, the relative reduction of levels of persister cells in the present study was possibly due to the transformation between persister and susceptible cells. Further research is needed to isolate native persisters from the biofilm using cell sorting methods and investigate their transformation into rapidly growing susceptible cells. Nevertheless this conversion was indeed incorporated in the mathematical model developed here.

In conclusion, this study demonstrates the tolerance of multispecies oral biofilms to withstand complete killing by levels of CHX treatment that substantially exceed those concentrations known to kill a proportion of bacterial cells within biofilms. This tolerance might be explained by the presence of persister cells within the biofilm population that leads to biofilm recovery over time. Traditionally, the post-antibiotic effect of CHX suggests that the antimicrobial activity of this compound would continue over long periods of time. Here, we show that bacteria in biofilms eventually re-grow despite the presence of residual levels of CHX. Quantitatively, our mathematical model agrees well with the experimental finding. The model prediction is based on the assumption of the existence of persisters in the biofilm. From a clinical perspective, these results have implications for determining the optimal interval between CHX treatments of oral biofilms, and reveal the inability of even the most active CHX formulation tested to completely wipe out the multispecies oral biofilm. Also, the data presented here are consistent with the suggestion that recalcitrant oral biofilms may be responsible for difficult-to-treat root canal infections. Our findings here may relate to different types of oral biofilms and other biofilm-related phenomena. It is hence necessary to identify compounds that synergize with CHX to prevent biofilm re-growth. The results presented here perhaps cannot be directly extrapolated to multispecies oral biofilms in the root canal in human patients, but they serve as a guide for future investigations in the field of endodontic and oral biofilms. The result confirms that mathematical modeling is a useful tool for analyzing and predicting the effects of antimicrobial agents in bacterial biofilms, and further understanding the role that persister cells play in resistance to antimicrobials.

## Materials and Methods

### Experiment Development

Sterile hydroxyapatite (HA) discs (9.7 mm diameter by 1.5 mm thickness; Clarkson Chromatography Products, Williamsport, PA) were used as the biofilm substrate. The HA discs were coated with bovine dermal type I collagen (10 μg/mL collagen in 0.012 N HCl in water) (Cohesion, Palo Alto, CA) as previously described^24^,^25^,^26^,^28^. Subgingival plaque was collected and suspended in Brain Heart Infusion broth (BHI, Becton Dickinson, Sparks, MD, USA). This work was approved by the Clinical Research Ethics Board of the University of British Columbia, Canada (H12-00907), written informed consent was obtained^26^. The discs were incubated under anaerobic conditions (AnaeroGen, OXOID, Hampshire, UK) at 37˚C for 21 days; fresh medium was changed once a week. After 21 days of anaerobic incubation in BHI broth, specimens were then immersed in 2 mL of either 2% CHX freshly prepared from 20% stock solution (Sigma Chemical Co, St Louis, MO) or CHX-Plus (<2% CHX gluconate solution with surface modifiers) (Vista Dental Products, Racine, WI) for 1, 3, or 10 minutes. The inactivator contained 1g α-Lecithin, 3 mL Tween 80, and 100 mL distilled water. After treatment, inactivator (2 mL) was applied for 60 s to inactivate chlorhexidine in half of the specimens. All specimens were then allowed to recover for the following eight weeks with the addition of fresh BHI broth once a week. Twelve specimens tested with saline for corresponding time periods were used as controls. For these specimens, the medium was changed once a week for eight weeks. Samples for CLSM for viability staining were collected immediately and 1, 2, 3, 5, 7 and 8 weeks after the exposure to the medicaments. Samples for scanning electron microscopy (SEM) were collected immediately and 1, 3, 5 and 8 weeks after the treatments.

#### SEM Examination

Two biofilm specimens in each group were cut into four sections and measured for biofilm thickness by SEM (Helios Nanolab 650, FEI, Eindhoven) operating at 10 kV. The specimens were washed with phosphate-buffered saline for 5 minutes. Fixation was performed by adding 2.5% glutaraldehyde for 30 minutes and 1% osmium tetroxide (OsO_4_) for 1 hour. The specimens were dehydrated by increasing concentrations of ethanol, dried by using a critical point drier (Samdri-795; Tousimis Research Corporation, Rockville, MD), and sputter-coated with gold-palladium in a vacuum evaporator (Hummer VI; Technics West Inc, Anaheim, CA). The thickness of three random areas of the biofilm on each piece was measured using ImageJ software (ImageJ 1.34n; National Institutes of Health, Bethesda, MD).

#### CLSM Analysis

The biofilm discs for CLSM imaging were rinsed in 0.85% physiological saline for 2 minutes to remove the culture broth. Two biofilm discs were examined for each time period. Five random areas of the biofilm on each disc were scanned (10 areas in each group; a total of 910 area scans for the whole study). To distinguish between live and dead cells, LIVE/DEAD *BacLight* Bacterial Viability kit L-7012 for microscopy and quantitative assays (Molecular Probes, Eugene, OR), which contained separate vials of the two component dyes (SYTO 9 and propidium iodide in 1:1 mixture) in solution, was used to stain the biofilm, following the manufacturer’s instructions. The excitation/emission maxima for these dyes are 480/500 nm for the SYTO 9 stain and 490/635 nm for propidium iodide. Fluorescence from the stained cells was viewed using a CLSM (Nikon Eclipse C1, Nikon Canada, Mississauga, ON), equipped with Nikon Image analysis software. Simultaneous dual channel imaging was used to display green and red fluorescence.

CLSM images of the biofilms were acquired by the EZ-C1 v. 3.40 build 691 software (Nikon) at a resolution of 512 × 512 pixels. Individual biofilm images covered an area of 1.64 mm^2^ per field of view. The mounted specimens were observed using a 10 x lens. Confocal LIVE/DEAD images were analyzed and quantitated using the Imaris 7.2 software (Bitplane Inc, St Paul, MN). The volume ratio of green fluorescence (live cells) to green-and-red fluorescence (live and dead cells) indicated the portion of live cells in the biofilm at each time. The method has been described in detail in previous studies^24^,^25^,^26^,^28^. The results were analyzed using Univariate ANOVA followed by post hoc analysis at a significance level of *P* < 0.05.

### Mathematical Model Formulation

#### Model assumptions and derivation

A mathematical model was developed to describe the reactive kinetics in the oral biofilm. The model parameters were calibrated against experimental data obtained from our previous study (biofilms treated by CHX for 1, 3 and 10 minutes)^26^ and current experiment (biofilms recovery over time after exposure to CHX). In this model, the bacteria were modeled in three phenotypes: the susceptible (the ones that were susceptible to antimicrobial agents), the persister (the ones that were persistent to antimicrobial agents), and the dead bacteria. Their volume fractions were denoted, respectively, as S, P and D. Besides the bacteria, the volume fractions of the EPS and solvent were also calculated, which are denoted by E and T, respectively. We assumed the material mixture constituting the biofilm was incompressible, i.e.


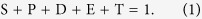


Experimental evidence showed that bacteria would undergo a lag phase once transferred into new circumstances. To account for this physiological and regulatory lag process of biofilm formation, we introduced a functional component named growth factor in this model, to regulate cell proliferation. It represents the necessary signal molecules or proteins, or extra-cellular DNA produced in the lag phase^61^,^62^,^63^, which affects cell proliferation as well as synchronization of quorum sensing molecules in a later stage.

Molecules of the growth factor, nutrient, antimicrobial and quorum sensing component are small compared with the other components comprising the biomass. Hence, we disregard their mass in the model. We denote the concentration of nutrients, QS molecules, antimicrobial agents and growth factors as C, H, A and Q, respectively. Although their mass is neglected, their chemical and biological effects are prominent and thereby retained.

#### Reactive equations for different phenotypes of bacteria

We assume both susceptible bacteria and persister bacteria can proliferate following a logistic model with the growth rate regulated by the growth factor^64^, antimicrobial agents^65^ and nutrient. It is hypothesized that the susceptible and the persister cells can convert into each other^66^. The rate of conversion from susceptible to persister cells is denoted as *b*_*sp*_ and the inverse conversion rate as *b*_*ps*_. In addition, we denote the natural death rate for the susceptible bacteria as *r*_*bs*_. The antimicrobial agent kills susceptible and persister cells at different rates, which are denoted as *c*_3_ and *c*_12_, respectively. The killing rate for persisters is presumably very low^32^ (which is the reason why they are named as persisters). Summarizing the mechanisms assumed above, we propose the reactive kinetic equation for susceptible bacteria and persisters as follows


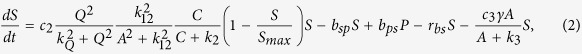



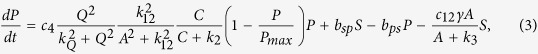


where *S*_*max*_ is the carrying capacity for the susceptible bacteria and *k*_12_, *k*_3_ are the half salutation rate for hill type reactive kinetics. Here γ is the slow-penetration factor (a relaxation parameter to represent the effect of spatial diffusion in the spatially homogeneous system), which is proposed in the Hinson model as follows^67^


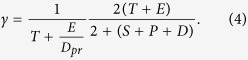


The prefactors in the form of Hill-type functions are postulated and calibrated to achieve the best fitting result.

The concentration of the dead bacteria is governed by the following reaction equation:


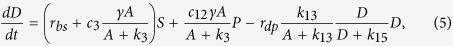


where, on the right hand side, the first two terms are the growth terms due to the death of susceptible and persister cells, respectively, and the last term represents the breakdown of dead bacteria into EPS and solvent components due to cell lysis^68^ as well as a spatial diffusion effect. Here *r*_*dp*_ is the maximum break down rate of dead bacteria, and *k*_13_, *k*_15_ are the half salutation rates of the Hill type switch function.

#### Reactive equation for EPS production

For EPS production, we assume that live bacteria produce EPS with a growth rate affected by the concentration of nutrient and quorum-sensing molecules^69^,^70^,^71^. The mechanism is effectively modeled by the Hill type kinetic equation. Since quorum sensing molecules affect the gene expression of the bacterial cells, they regulate effectively the EPS production by both susceptible and persister cells. Besides, we require that the rate of EPS production be reduced as the concentration of EPS increased. The dead bacteria can disintegrate to shed their surface-attached EPS^68^, which contributed to the second term in the following reactive equation. The reactive equation is given as follows:


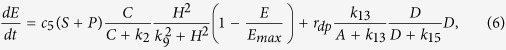


where the first part represents the gain of EPS due to the live bacteria and the second part represents that gain from the dead bacterial conversion. Here *c*_5_ is the maximum EPS production rate and *k*_2_, *k*_9_ are the half salutation rates in the Hill functions.

#### Reactive equations for functional components

For nutrient and antimicrobial agent concentration, the monod model is appropriate. For the QS molecules and growth factors, however, their production depended on the concentration of the susceptible bacteria while, in the meantime, saturate at a maximum level in the presence of a large number of the QS molecules or growth factors. We adopt the notion that the QS molecule facilitates the EPS production, which has been widely discussed in literature, and its population growth is affected by the growth factor in a Hill’s dynamics and bounded above by a threshold concentration. The Hill’s type growth depending on Q in the growth rate of H is hypothesized to show that the effect of the growth factor is quadratic in Q at a low concentration and saturates at a high one. So far, there is little evidence showing the influence of QS molecules on growth factor. Thus, we assume dQ/dt is independent of H in the model. For these molecules, we thus propose a coupled system of reactive equations:


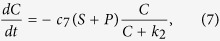



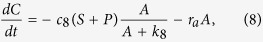



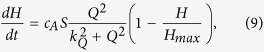



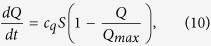


where *c*_7_,*c*_8_ are the maximum consumption rate for nutrient and antimicrobial agents, respectively, *c*_A_, *c*_q_ are the maximum production rate for QS molecules and growth factors, respectively, *k*_2_, *k*_8_ are the half salutation rates, and *r*_*a*_ is the natural decay rate, indicating that the antimicrobial agents would lose effectiveness in time due to spatial diffusion as well as losing efficacy. Here, *H*_*max*_, *Q*_*max*_ are the saturation level for QS molecules and growth factors, respectively. The QS kinetic is strongly coupled to the EPS growth while the growth factor impacts the reproduction of the susceptible cells. Without the couplings, we have not been able to fit the model to the experimental data.

#### Summary of the governing equations

In summary, the coupled ordinary differential equations for biofilm model are given as follows


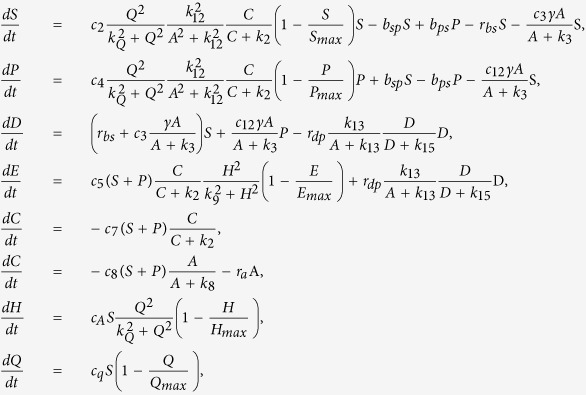


where


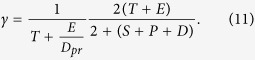


Note that we use *c*_0_, *d*_0_, *H*_*max*_, *Q*_*max*_ and *t*_0_ to non-dimensionalize the system. Thus, in the discussion of the model, the concentrations of non-dimensionalized functional molecules are all range from 0 and 1. We solve the dynamical system using an ODE solver in Matlab. The model parameters are first calibrated against the experiments alluded to earlier. This model can be readily extended to include spatial convection and diffusion to describe any heterogeneous effects in the biofilm.

## Additional Information

**How to cite this article**: Shen, Y. *et al.* Experimental and Theoretical Investigation of Multispecies Oral Biofilm Resistance to Chlorhexidine Treatment. *Sci. Rep.* **6**, 27537; doi: 10.1038/srep27537 (2016).

## Figures and Tables

**Table 1:** Values of the model parameters.

hbn	Description	Value	Unit	Reference
*c*_0_,*H*_*max*_,*d*_0_,*Q*_*max*_	Characteristic molecule concentration	8.24 × 10^−3^	kg/m^3^	28
*c*_2_	Maximum growth rate for the susceptible	3 × 10^−6^	*s*^−1^	
*c*_3_	Maximum death rate for the susceptible	6.5 × 10^−2^	*s*^−1^	
*c*_4_	Maximum growth rate for the persister	3 × 10^−7^	*s*^−1^	
*c*_12_	Maximum death rate for the persister	6 × 10^−4^	*s*^−1^	
*c*_5_	Maximum EPS production rate	3.5 × 10^−3^	*s*^−1^	
*c*_7_,*c*_8_	Maximum nutrient consumption or antimicrobial consumption rate	1.0 × 10^−7^	*s*^−1^	
*c*_*A*_	QS molecule production rate	6 × 10^−7^	*s*^−1^	
*c*_*q*_	Growth factor production rate	1.0 × 10^−5^	*s*^−1^	
*r*_*a*_	Decaying rate of effective antibiotics	1.0 × 10^−6^	*s*^−1^	
*b*_*sp*_,*b*_*ps*_	Conversion rate between the persister and the susceptible	1.5 × 10^−7^	..	4
*r*_*bs*_	Natural death rate of susceptible bacteria	2.0 × 10^−7^	*s*^−1^	
*r*_*dp*_	Dead bacteria recycling rate into EPS	2.2 × 10^−6^	*s*^−1^	
*D*_*pr*_	Hinson constant	0.007		29
*k*_3_	Monod constant	3.5 × 10^−3^	kg/m^3^	4
*k*_2_,*k*_8_	Monod constant	3.5 × 10^−4^	kg/m^3^	30
*k*_9_	Monod constant	6.6 × 10^−3^	kg/m^3^	31
*k*_12_	Monod constant	6.0 × 10^−7^	kg/m^3^	
*k*_*Q*_	Monod constant	2.5 × 10^−3^	kg/m^3^	
*S*_*max*_	Carrying capacity for the susceptible bacteria	0.08		
*P*_*max*_	Carrying capacity for the persister bacteria	0.018		
*E*_*max*_	Carrying capacity for EPS	0.15		

Some values of the model parameters or their orders of magnitude are adopted from the literature, whose sources are referenced. Most of the model parameters are obtained by fitting the model to the experiment results in^26^ and^32^.
